# Prevalence and Risk Assessment of Aflatoxin in Iowa Corn during a Drought Year

**DOI:** 10.1155/2023/9959998

**Published:** 2023-11-20

**Authors:** Emily H. Branstad-Spates, Erin L. Bowers, Charles R. Hurburgh, Philip M. Dixon, Gretchen A. Mosher

**Affiliations:** ^1^Department of Agricultural and Biosystems Engineering, Iowa State University, Ames, Iowa 50011, USA; ^2^Department of Statistics, Iowa State University, Ames, Iowa 50011, USA

## Abstract

Warm temperatures and drought conditions in the United States (US) Corn Belt in 2012 raised concern for widespread aflatoxin (AFL) contamination in Iowa corn. To identify the prevalence of AFL in the 2012 corn crop, the Iowa Department of Agriculture and Land Stewardship (IDALS) conducted a sample of Iowa corn to assess the incidence and severity of AFL contamination. Samples were obtained from grain elevators in all of Iowa's 99 counties, representing nine crop reporting districts (CRD), and 396 samples were analyzed by IDALS using rapid test methods. The statewide mean for AFL in parts per billion (ppb) was 5.57 ppb. Regions of Iowa differed in their incidence levels, with AFL levels significantly higher in the Southwest (SW; mean 15.13 ppb) and South Central (SC; mean 10.86 ppb) CRD (*p* < 0.05) regions of Iowa. This sampling demonstrated high variability among samples collected within CRD and across the entire state of Iowa in an extreme weather event year. In years when Iowa has AFL contamination in corn, there is a need for a proactive and preventive strategy to minimize hazards in domestic and export markets.

## 1. Introduction

More than 300 mycotoxins produced by various fungal species have been identified and characterized. Among these, aflatoxins (AFL) are currently recognized as the most significant in terms of their impact on food and feed safety. AFL is a class of mycotoxins produced primarily by *Aspergillus flavus* and *Aspergillus parasiticus* fungi (CAST, [1]). They are found worldwide in various foods and major feed ingredients for production animals. Over 100 countries have established regulations or guidance to limit AFL in the food and feed supply (FAO, [2]). The US Food and Drug Administration (FDA) has established a 20 ppb regulatory action level for AFL in corn for use in general commerce (with unknown end use) (FDA, [3]). Corn contaminated above this action level up to 300 ppb has limited feed use by specific livestock (Table 1). Further, corn contaminated above 300 ppb is considered adulterated for any use in all US states.

The US livestock industry must exercise caution and control AFL in animal feed to prevent negative impacts on animal health and productivity. Cattle are the most tolerant species to AFL in feed, but excessive dietary exposure may decrease reproductive efficiency and milk production and lead to the development of liver lesions (Jouany & Diaz, [4]). The action level for dairy cattle is reduced relative to beef cattle because a toxic metabolite of AFL (aflatoxin M_1_; AFM_1_) is transmitted to cow's milk at a rate of up to 6–27% of the ingested AFL (Branstad, [5]; Frobish et al., [6]; Rodrigues, [7]). Limiting dairy cattle exposure through feed effectively mitigates the risk of human exposure through the consumption of dairy products. Adverse effects of AFL in swine include reduced growth rates, liver damage, and bleeding. Poultry experience reduced egg production, embryo losses, and feed efficiency due to AFL consumption (Monson et al., [8]; Rawal et al., [9]; Robens & Richard, [10]).

Accurately monitoring and quantifying AFL contamination in bulk grain and feed ingredients is important to food and feed safety but can be difficult. AFL is unevenly distributed in fields, storage bins, and grain lots in corn, yet it can occur at high concentrations in individual kernels. Shotwell et al. [11] reported 207,000 ppb aflatoxin B_1_ in an individual kernel of southern corn in 1969 and 1970. Assuming a kernel weight of 0.3 g, as few as eight individual kernels contaminated at this level in a bushel of corn (56 pounds) would result in the mean contamination of that bushel reaching the 20 ppb action level. Such highly contaminated kernels are rare in US corn; kernels are more likely to be contaminated with a range of AFL levels. Johansson et al. [12] estimated that, in a 20 ppb AFL-contaminated corn lot, approximately 6 in 10,000 kernels are contaminated. Detection is highly dependent on a representative sampling of grain lots. Even with representative sampling, contaminated kernels may be missed in large lots.

The USDA has prescribed sampling methods for various grain transportation units to acquire the most representative sample possible (USDA-FGIS, [13]), yet the practical feasibility of these sampling methods is limited. In the short three- to four-week harvest period from September to October, growers deliver large volumes of grain to elevators and other processors. Receiving rates of 100-400 semitruck loads daily, each carrying up to 900-950 bushels, are common (Hurburgh, [14]; Laux et al., [15]). Performing “rapid” AFL tests before each grain load acceptance requires additional processing time and labor. This added time, typically 10-15 minutes per test, would result in harvest delays as trucks wait on scales to be dumped at the elevator, possibly leading to a grower's choice to sell their grain at other locations (Robens & Cardwell, [16]). For this reason, the incentive for businesses to implement thorough sampling and testing techniques is low. The high-volume throughput of commercial bulk grain during harvest and postharvest handling limits the practicality of representative sampling and testing of every incoming grain load.

Further, postharvest handling is only part of the AFL mitigation problem. AFL occurrence is highly dependent on weather and climate. AFL-producing *Aspergillus* fungi occur naturally in soil and are most prevalent in warm to hot, dry conditions. The climatic preference of AFL-producing fungi results in an annual risk for AFL contamination in southern US corn. AFL contamination in the US Corn Belt, comprising the states of Illinois, Indiana, Iowa, Michigan, Minnesota, Missouri, Ohio, and Wisconsin, where most of the US corn crop is produced, is less common as temperature patterns and rainfall timing are typically sufficient to impede the growth of *Aspergillus* (Robertson, [17]). Infrequent AFL outbreaks in the Corn Belt corn have been associated with drought years in the Midwest US. In Iowa, historically the top corn-producing state in the US with 1.88 billion bushels of corn in 2012, AFL outbreak events of varying degrees of severity have been documented, including 1983, 1989, 2005, and 2012 (Mitchell et al., [18]; Russell et al., [19]; Schmitt & Hurburgh, [20]; Tuite et al., [21]; USDA-NASS, [22]; Zuber & Lillehoj, [23]). Predicting these events is difficult, which limits *in situ* study design. Modeling efforts have been made in Illinois and Iowa, but the transferability of the models has not been demonstrated among geographic regions (Branstad-Spates et al., [24]; Castano-Duque et al., [25]). To be effective, growers' investments in preventive measures must be made before a drought occurs and before the possibility of AFL detection. These investments are difficult to justify when outbreaks are atypical. While AFL is not an annual risk in Iowa or other Corn Belt states, outbreak events in these and other high-production regions endanger a large portion of the total US crop, compromise the overall security of the US corn crop, and incite a corresponding negative impact on the grain market (Mitchell et al., [18]).

**Figure 1:**
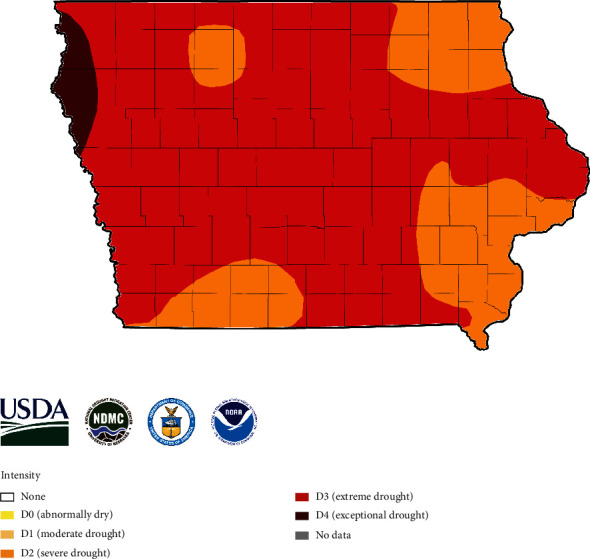
National Drought Mitigation Center map for Iowa on October 2, 2012, during the harvest period.

In 2012, as the National Drought Mitigation Center [26] reported, most of Iowa suffered moderate to extreme drought starting in July and continuing through harvest (Figure 1). The drought, accompanied by high temperatures, created a favorable environment for *Aspergillus* fungal pathogens, with a corresponding increase in the risk of an AFL outbreak (cli-MATE, [27]). A statewide sampling was conducted to estimate the distribution of AFL contamination throughout Iowa and to determine the overall burden of AFL contamination in the state's corn supply.

## 2. Materials and Methods

**Figure 2:**
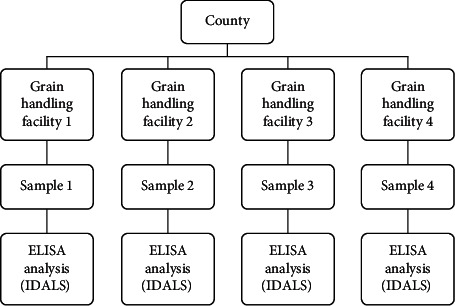
Sampling schematic for collecting corn samples from each Iowa county.

A statewide sampling for AFL contamination in the 2012 Iowa corn harvest was conducted by the Iowa Department of Agriculture and Land Stewardship (IDALS). Four corn samples were collected during the corn harvest from 4 different grain-handling facilities in each of Iowa's 99 counties.  Figure 2 shows the sampling schematic used in each county. If it was not possible to collect samples from four unique grain-handling facilities per county, samples were taken at a different time from a repeat location(s). Samples were collected from the scale-house probe grain depositories, which is the grain that has been collected from incoming grain loads for grading purposes. Thus, the samples analyzed in the current study represent mixtures of the loads received on the day they were collected, with representative moisture contents from the grain handling organizations. Samples were not dried at the grain handling facility. Sample sizes ranged from 3 to 10 lbs. (1320 to 4460 g) of whole kernel corn, with a mean sample weight of 6.7 lbs. (3020 g).

Independent samples at IDALS were ground using a Romer Series II subsampling mill. Approximately 1/3 of the sample was collected from the original corn sample as a subsample. A test portion was selected from the 1/3 portion and analyzed using the AgraQuant ELISA Total Aflatoxin Assay (B_1_ + B_2_ + G_1_ + G_2_) (COKAQ1000 4-40 ppb) (Romer Laboratories, Union, MO, USA), according to manufacturer instructions. The assay has a detection limit of 3 ppb (Romer Laboratories, Union, MO, USA). This analysis method detected and reported the sum of aflatoxins B_1_, B_2_, G_1_, and G_2_.

IDALS analyzed 396 corn samples by enzyme-linked immunoassays (ELISA). Mean log-transformed AFL levels for each crop reporting district (CRD) (Table 2) were estimated using maximum likelihood (Helsel, [28]). The median AFL levels were estimated by exponentiating the mean log-transformed values, and the mean AFL levels were estimated by

(1) $$\begin{array}{c} \text{mean }{\text{AF}}_{i}=\exp\left({\hat{\mu }}_{i}+\frac{{\overset{\wedge }{\sigma }}^{2}}{2}\right), \end{array}$$

where ${\hat{\mu }}_{i}$ is the estimated mean log AFL level for crop reporting district *i* and ${\overset{\wedge }{\sigma }}^{2}$ is the estimated variance. A likelihood ratio test was used to test the null hypothesis of no difference in regions' mean levels. SAS PROC LIFEREG, version 14.3, was used for these computations (SAS Institute Inc., Cary, NC, USA).

## 3. Results

**Figure 3:**
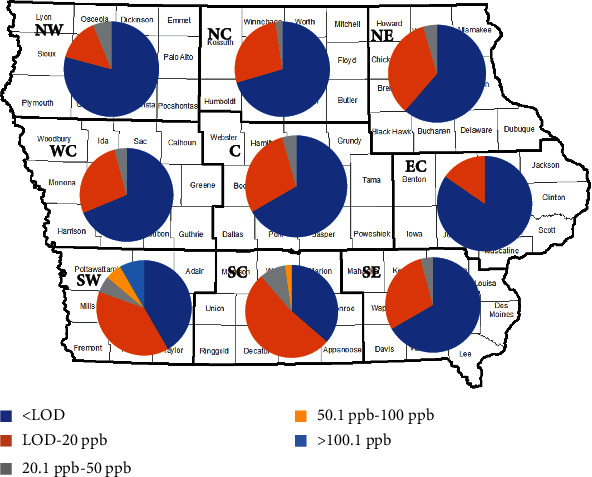
Nine Iowa crop reporting districts (CRD) and aflatoxin distributions within each district (*n* = 396).

The statewide mean of all AFL test results was 5.57 ppb (*n* = 396) from all the corn samples. There was evidence for differences in the overall mean AFL levels among the nine CRDs (*p* < 0.01). Corn from Southwest (SW) Iowa had the highest AFL level among the districts, with a mean 15.13 ppb total AFL (*p* < 0.01). This indicates that, on average, corn from this district was within range of the acceptable limit for use in general commerce. However, a mean greater than 15 ppb edges closer to the lowest threshold of 20 ppb. SW corn had significantly higher AFL contamination than all other districts except South Central (SC) (mean 10.86 ppb; Table 2). There were no significant differences among the remaining seven districts, including East Central (EC), which had the lowest mean AFL contamination (mean 1.78 ppb; Table 2).  Figure 3 showcases the nine CRDs and the distribution of AFL in the state of Iowa.

The overall mean AFL level by CRD obtained through this sampling was combined with production in bushels as reported by the US Department of Agriculture National Agricultural Statistics Service (USDA-NASS) for each corresponding district to calculate the contribution of AFL contamination, by weight, of each district to the total contamination in the state corn supply (Table 3; USDA-NASS [29]). Using all samples (*n* = 396) and weighting for production differences by CRD, the state average for AFL in the 2012 Iowa corn crop at harvest was 4.38 ppb, compared to the arithmetic mean (unadjusted) of 5.57 ppb. The SW region had one of the lowest values for corn production at 6.91% statewide; however, it had the highest AFL contamination when adjusted with 23.85% of the statewide contribution. On average, the SW region has lower corn production than other Iowa regions. In contrast, the SC region had the second-highest overall mean at 10.86 ppb. With only 2.44% of AFL contribution in the SC region, when adjusted for statewide contribution, it increased to 6.05%.

## 4. Discussion

Researchers and industry professionals understand most theoretical aspects of mycotoxin risk (Branstad-Spates, [30]). However, translating this knowledge into a practical, feasible management system for a high-volume production area is more challenging. To comply with action levels and ensure the future marketability of grain, grain handlers and processors may employ some form of mycotoxin testing strategy for incoming grain. This action is often inconsistent for several reasons. Mycotoxin contamination is largely a function of climate and weather, so the risk changes yearly, even between years of ostensibly similar conditions (Medina et al., [31]). Mycotoxins are not completely avoidable, even with good agricultural and management practices. AFL often occurs in localized areas of a field (rather than uniformly distributed throughout the commodity), their concentration is not uniform among grain kernels, and they can occur at very high levels in single kernels (Shotwell et al., [11]). The inconsistency has confounded systematic interpretation, resulting in the sporadic application of inefficient and ineffective strategies for mycotoxin management. This is especially true at initial entry points for grain into the commodity stream and at grain handling firms in low-incidence but high-impact areas like the Corn Belt states.

The heterogeneous nature of AFL contamination is well recognized, and best management practices have been prescribed for individual lot sampling (European Commission, [32]; GIPSA-FGIS, [33]; Whitaker et al., [34]). Using these procedures could mitigate a significant portion of the risk annually by more frequently identifying and diverting contaminated lots before they enter general commerce. However, individual lot sampling strategies are inconsistently adopted because they are labor-intensive and cost- and time-prohibitive in high-throughput operations, where most commodity corn is received into general commerce (Whitaker et al., [34]). Decisions on grain lot acceptance or rejection are made at harvest in 1-2 minutes. Spending 15 to 30 minutes sampling, grinding, subsampling, and running a rapid test on each incoming lot is not feasible. Depending on the lot size, labor, and materials, it can easily exceed $20 per test, equivalent to 3-5 cents per bushel (Robens & Cardwell, [16]). The cost is difficult to justify in low-margin commodity grain industries if the grain is expected to generally meet tolerance levels. Volume and time limitations result in the application of sporadic testing of individual loads in years when conditions are conducive to AFL formation, but the use of organized mycotoxin testing strategies in these settings has not been normative.

The Illinois Department of Agriculture also performed a statewide mycotoxin sampling in 2012, and 75 of the 400 corn samples collected (18.8%) tested at over 20 ppb AFL in Illinois. These “hot spots” were not isolated to one area. They were found in over half of the counties in the state (Illinois Department of Agriculture, [35]). Illinois was fourth in corn production among US states in 2012, and Iowa and Illinois corn production was nearly 30% of the total US production (USDA-NASS, [36]). Of the samples tested throughout Iowa in the current study, 24 out of 396 (6.10%) tested above 20 ppb AFL. Therefore, as a whole, Iowa did have fewer high-contamination samples than Illinois. Only the EC region had no AFL test result above 20 ppb. The remaining regions all had evidence of AFL contamination “hot spots” in incoming grain that could prove problematic for food and feed safety.

Under normal circumstances, AFL “hot spots” are naturally mitigated at commercial grain handling facilities when grain lots are commingled in bins at receiving and when the grain is mixed to achieve contractual specifications for other grade factors (Laux et al., [15]). However, there have been documented years, even in weather conditions unfavorable to producing AFL, where a few samples may be over the 20 ppb regulation. Additional homogenization may occur in processing steps if it is present. If sufficient blending is not achieved, sporadic test results over 20 ppb will occur at later stages of the market chain, where the challenge of isolation and diversion increases (Shi et al., [37]; Shotwell, [38]). AFL hot spots in grain can potentially result in toxicity in exposed individuals, be they humans or animals. Human exposure to AFL contamination is not typically a problem in the US, where consumption of corn and corn-based products is low and product testing is thorough. It can be problematic in populations for which corn is a dietary staple and for subsistence farmers, especially in regions with climates and weather regularly suitable for AFL. Pet and livestock exposure is a more pressing concern in the US, as corn and other mycotoxin-prone ingredients are common staple ingredients (Aquino & Corrêa, [39]; Boermans & Leung, [40]). Pet food recalls for AFL contamination occurred in 2013, likely due to AFL contamination in the 2012 corn crop (USDA-HHS, [41]).

High-volume production regions significantly impact the overall US corn supply's quality, safety, and utility. When AFL contamination occurs in Iowa and other Corn Belt states, a significant portion of the total US crop is at risk. A small-scale example of the situation can be taken from this study: the Central (C) region of the state had a mean 3.55 ppb total AFL in the current study but was the second-highest corn production region in the state that year, making it the second only to SW Iowa in total AFL contribution (by weight) at 13.26%. Another illustration from the current study: less than 7% of the corn harvested statewide in 2012 was produced in the SW region, but the AFL contamination in that region (mean 15.13 ppb) comprised 23.85% of the statewide AFL contamination by weight. The SC district had the second-highest mean AFL level (mean 10.86 ppb), but this was the least productive region in the state in 2012, contributing only 2.44% of total state production in bushels and, subsequently, only 6.05% of the AFL (by weight).

On a national scale, southern US corn growers face an annual risk of AFL contamination because of regular climatic conditions favorable for *Aspergillus flavus*. Yet, the US South grows less corn; however, these states grow corn to keep feed costs down for feedlot and poultry production. In 2012, corn production in Texas totaled nearly 200 million bushels (USDA-NASS, [42]). The amount equals about 10% of that produced in Iowa and less than 2% of total US production. Large-volume production in the low-risk Corn Belt typically mitigates the nation's overall risk. However, in 2012, Iowa could only have as much contamination as Texas does yearly if its AFL mean contamination level was ten times greater (approximately 33.7 ppb). In years when Corn Belt states face even a moderate AFL risk, the interdependence among corn-producing states is a fragile balance. At risk is the ability to secure a national corn supply with AFL concentrations low enough to fulfill domestic and export market demands safely and sufficiently. For these reasons, there is a need for a proactive and preventive strategy to manage AFL in low-incidence but high-volume and high-impact areas.

## 5. Conclusion

Temperatures and drought conditions were conducive to producing AFL in the US Corn Belt in 2012, leading to contamination events in the nation's leading corn-producing state. Samples obtained from IDALS in Iowa's 99 counties aggregated into nine CRDs were analyzed (*n* = 396) to evaluate AFL prevalence rates. The overall statewide mean for AFL was 5.57 ppb; however, CRDs differed in contamination levels, with the Southwest region being the highest for AFL at 15.13 ppb. This study showcased high variability among AFL prevalence rates in an extreme weather outbreak in Iowa.

## Figures and Tables

**Table 1:** FDA^1^ action levels for aflatoxin in animal feeds.

Class of animals	Feed	Aflatoxin level
Finishing beef cattle	Corn and peanut products	300 ppb
Beef cattle, swine, or poultry	Cottonseed meal	300 ppb
Finishing swine over 100 lb.	Corn and peanut products	200 ppb
Breeding cattle and swine, mature poultry	Corn and peanut products	100 ppb
Immature animals	Animal feeds and ingredients (excluding cottonseed meal)	20 ppb
Dairy animals, animals not listed elsewhere, or unknown use (general market)	Animal feeds and ingredients	20 ppb

^1^US Food and Drug Administration, Action levels for poisonous or deleterious substances in human food and animal feed. US Department of Health & Human Services, Editor. 2000.

**Table 2:** Mean aflatoxin contamination (ppb) in corn by crop reporting district (CRD) in 2012 in Iowa. Mean results are reported for each CRD. The % of samples reported that tested above the 20 ppb aflatoxin limit and the % of samples that fell below the limit of detection (LOD).

Crop reporting district (CRD)	Total samples (*n*)	Overall mean (ppb)	*p* value^1^	SEM^2^	% of samples testing > 20 ppb total aflatoxin	% of samples testing below LOD (limit of detection)
Northwest	48	2.88	0.76	0.28	6.25	20.83
West Central	48	3.90	0.13	0.26	4.17	31.25
Southwest	36	15.13	<0.01	0.25	19.44	58.33
North Central	44	3.17	0.51	0.28	2.27	29.55
Central	48	3.55	0.25	0.26	4.17	33.33
South Central	44	10.86	<0.01	0.23	11.36	63.63
Northeast	44	4.25	0.06	0.26	4.55	38.64
East Central	39	1.78	0.26	0.35	0.00	15.38
Southeast	45	4.60	0.03	0.26	4.44	33.33
Total analyses	396	5.57				

^1^Main effect of CRD against other CRD in Iowa for AFL contamination levels (ppb). ^2^Greatest standard error is shown.

**Table 3:** Aflatoxin contribution by crop reporting district (CRD) adjusted for regional production differences in Iowa.

Crop reporting district (CRD)	2012 production (bushels)	Overall mean (ppb)	% of statewide production	Production adjusted aflatoxin contribution (kg)	% of statewide contribution of aflatoxin
Northwest	304,706,000	2.88	16.23	22.29	10.67
West Central	268,724,000	3.90	14.32	26.62	12.74
Southwest	129,711,000	15.13	6.91	49.85	23.85
North Central	282,405,000	3.17	15.05	22.74	10.88
Central	307,213,000	3.55	16.37	27.70	13.26
South Central	45,803,000	10.86	2.44	12.64	6.05
Northeast	236,675,000	4.25	12.61	25.55	12.23
East Central	190,753,000	1.78	10.16	8.62	4.13
Southeast	110,910,000	4.60	5.91	12.96	6.20

State-wide	1,876,900,000	5.57	100	208.97	100

Adjusted state-weight mean		4.38			

## Data Availability

The 2012 aflatoxin dataset presented in this study is confidential due to requests from collaborating sources and needs for ongoing Iowa State research building upon this database. Requests to access the datasets should be directed to GAM at gamosher@iastate.edu or the Iowa Department of Agriculture and Land Stewardship (IDALS).
